# The role of fibroblast growth factors in cell and cancer metabolism

**DOI:** 10.1002/1873-3468.70199

**Published:** 2025-10-23

**Authors:** Jessica Price, Chiara Francavilla

**Affiliations:** ^1^ Section of Medical Biotechnology, Department of Bioengineering and Biomedicine Technical University of Denmark Lyngby Denmark

**Keywords:** cancer, cell signaling, FGF, fibroblast growth factor receptor, homeostasis, metabolism, metastasis, receptor tyrosine kinase, therapy resistance

## Abstract

The fibroblast growth factor (FGF) family and the FGF receptors are ubiquitously expressed and regulate a plethora of cell signaling cascades during development, tissue and cell homeostasis, and metabolism. Dysregulated FGF signaling is associated with cancer and several genetic and metabolic disorders. As FGF signaling regulates all the key metabolic processes to maintain whole‐body homeostasis, there is an increasing focus on engineering FGFs as potential treatments for dysregulated metabolism. Within cancer, reprogramming of energy metabolism is a crucial step leading to tumorigenesis, metastasis formation, and resistance to therapy. FGF signaling dysregulation in cancer enables uncontrolled proliferation and survival and promotes therapy resistance and metastasis. However, the role of FGF signaling within cancer metabolism is not well understood. A better understanding of how FGF signaling affects the rewiring of cancer metabolism as well as tumorigenesis would provide novel avenues for discovering potential drug targets and biomarkers. Here, we discuss the role of paracrine, endocrine, and intracellular FGFs within metabolism as well as the current understanding of how FGF signaling contributes to rewired cancer metabolism.

## Graphical abstract

Fibroblast growth factor (FGF) signaling regulates crucial signaling cascades that promote cell proliferation, survival, and metabolism. Therefore, FGFs and their receptors are often dysregulated in human diseases, including cancer, to sustain proliferation and rewire metabolism. This review discusses the role of FGF signaling in cell metabolism and its contribution to cancer metabolism.

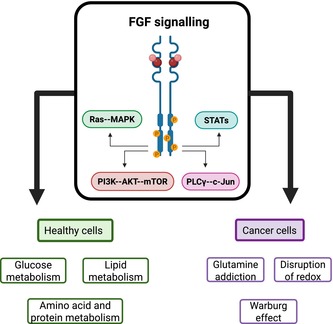

## Abbreviations

**ACC**, acetyl‐CoA

**ACE2**, angiotensin‐converting enzyme 2

**AKT**, protein kinase B

**AMP**, adenosine monophosphate

**AMPK**, AMP‐activated protein

**ATP**, adenosine triphosphate

**BAD**, BCL2‐associated agonist of cell death

**BAT**, brown adipose tissue

**cAMP**, cyclic adenosine monophosphate

**CAN**, central nervous system

**c‐Met**, cellular mesenchymal–epithelial transition factor

**ECM**, extracellular matrix

**EGFR**, epidermal growth factor receptor

**ER**, estrogen receptor

**ERK**, extracellular signal‐regulated kinase

**ETS1**, C‐ets‐1

**FASN**, fatty acid synthase

**FGF**, fibroblast growth factor

**FGF13‐AS1**, FGF13‐anti‐silencing 1

**FGFR**, fibroblast growth factor receptor

**FGFRL1**, fibroblast growth factor receptor‐like 1

**FPB1**, fructose‐1,6‐bisphosphatase 1

**FRS2α**, FGFR substrate 2α

**FXR**, farnesoid X receptor

**GAB1**, GRB2 associated protein 1

**GCGR**, glucagon receptor

**GF**, growth hormone

**GLUT**, glucose transporter

**GPCR**, G protein‐coupled receptor

**GRB2**, growth receptor‐bound protein 2

**GSH**, glutathione

**HGF**, hepatocyte growth factor

**HK**, hexokinase

**HPA**, hypothalamic–pituitary–adrenal

**HS**, heparin sulfate

**HSPG**, heparin sulfate proteoglycan

**i.c.v**., intracerebroventricular

**iFGF**, intracellular fibroblast growth factor

**Ig**, immunoglobulin

**IGF‐1**, insulin‐like growth factor 1

**IGFR**, insulin growth factor receptor

**JAK**, janus kinase

**JNK**, c‐Jun N‐terminal kinase

**LDH**, lactate dehydrogenase

**LKB1**, liver kinase B1

**LPL**, lipoprotein lipase

**MAPK**, mitogen‐activated protein kinase

**MASH**, metabolic syndrome‐associated steatohepatitis

**MEK**, mitogen‐activated protein kinase kinase

**mTOR**, mammalian target of rapamycin

**NADPH**, nicotinamide adenine dinucleotide phosphate

**NAFLD**, nonalcoholic fatty liver disease

**NCAM**, neural cell adhesion molecule

**NPY**, neuropeptide Y

**OXPHOS**, oxidative phosphorylation

**PD4D**, phosphodiesterase 4D

**PGC‐1α**, PPARγ coactivator protein‐1α

**PI3K**, phosphoinositide 3‐kinase

**PKCε**, protein kinase C epsilon

**PLC**, phospholipase C

**PPAR**, peroxisome proliferator‐activated receptors

**PPP**, pentose phosphate pathway

**PTB**, phosphotyrosine‐binding

**R6K**, ribosomal s6 kinase

**ROS**, reactive oxygen species

**RSK2**, ribosomal S6 kinase 2

**RTK**, receptor tyrosine kinase

**SEF**, similar expression to FGF

**SHB2**, Src homology 2

**SHP2**, src homology‐2 protein tyrosine phosphatase

**SPRY**, sprouty

**SREBP**, sterol regulatory element‐binding protein

**SRF**, serum response factor

**STAT**, signal transducer activator of transcription

**T1DM**, type 1 diabetes mellitus

**T2DM**, type 2 diabetes mellitus

**TAG**, triacylglycerol

**TCA**, the citric acid cycle

**UCP1**, uncoupling protein 1

**ULK1**, unc‐51 like autophagy activating kinase 1

**VEGF**, vascular endothelial factor

**VLDL**, very low‐density lipoprotein

**WAT**, white adipose tissue

### Receptor tyrosine kinases

Receptor tyrosine kinases (RTKs) are a large family of widely expressed transmembrane receptors that catalyze phosphorylation of target proteins to regulate key cellular processes including proliferation, metabolism, motility, and survival [1, 2, 3]. RTKs have a well‐conserved structure consisting of an extracellular region that contains at least one ligand‐binding domain, a single transmembrane helix, and an intracellular region with several catalytic tyrosine kinase domains [3, 4]. Ligand binding induces dimerization of receptors in the membrane and stabilizes an active conformation by stimulating the release of the *cis* autoinhibition and subsequent *trans* autophosphorylation of the tyrosine kinase domains within the cytoplasmic region [1, 5]. Intracellular signaling proteins, containing Src homology 2 (SHB2) or phosphotyrosine‐binding (PTB) domains, are then recruited to the phosphorylated receptor and activated [6, 7]. These in turn activate other proteins within key signaling cascades, such as phosphoinositide 3‐kinase/protein kinase B (PI3K/Akt), mitogen‐activated protein kinase (MAPK), phospholipase C (PLCγ), and Janus kinase/signal transducer activator of transcription (JAK/STAT) pathways that control cell proliferation, differentiation, survival, metabolism, and migration [1, 3, 8].

**Fig. 1:**
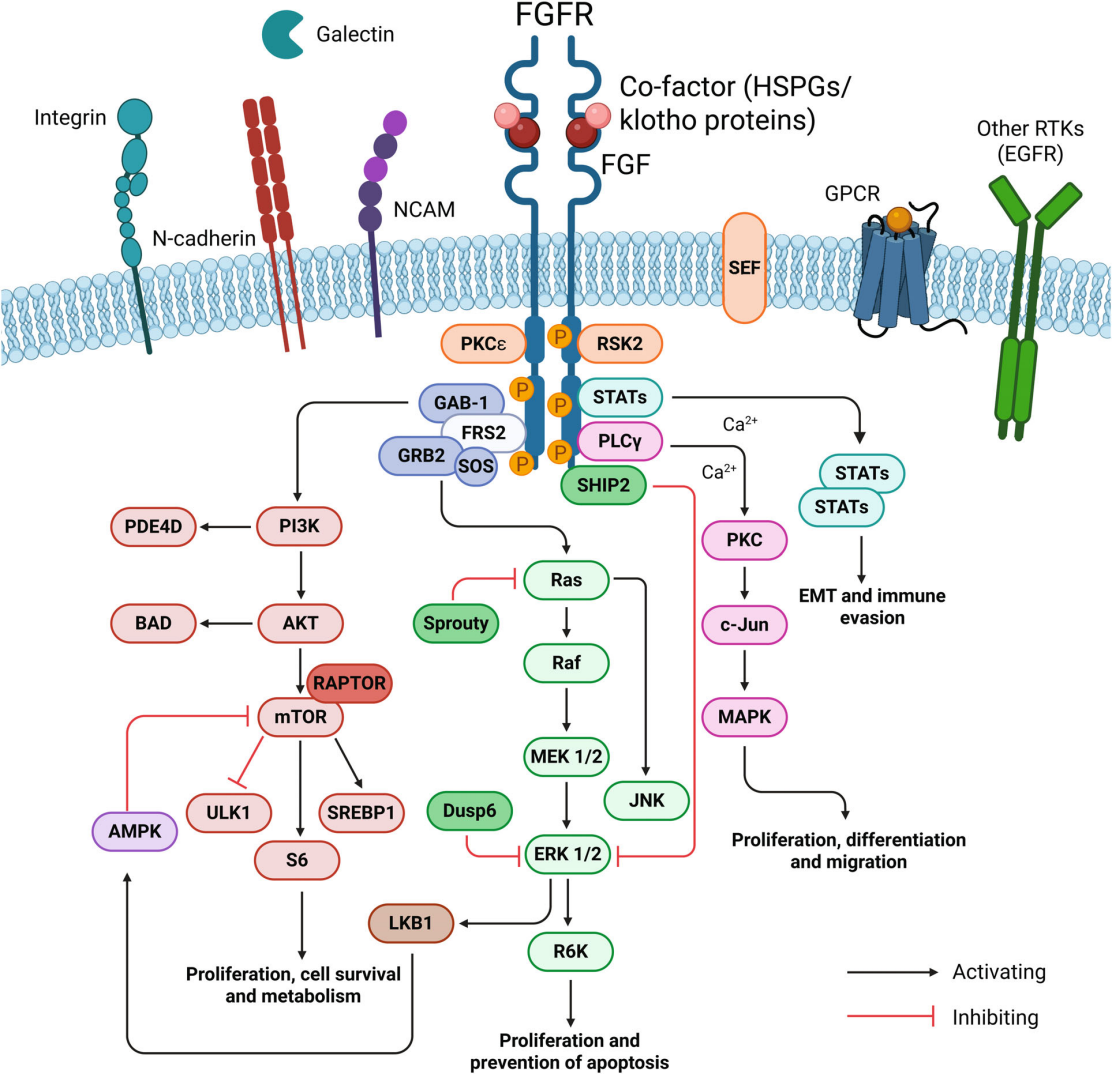
Schematic showing the signaling pathways activated downstream of FGF/FGFR, including noncanonical signaling partners such as N‐cadherin, neural cell adhesion molecule (NCAM), integrins, galectins, G protein‐coupled receptors (GPCR), and other RTKs such as EGFR [20, 40, 265]. Created with BioRender (KA28QW9QSM).

Dysregulation of RTK signaling has been implicated in the onset and progression of cancer as well as within various metabolic diseases and genetic disorders [3, 4, 9, 10, 11]. In this review, we will focus on one family of RTKs, the fibroblast growth factor receptors (FGFRs) and their ligands FGFs, as they play crucial roles not only throughout embryonic development and organogenesis, but also in adult tissue homeostasis, including the regulation of cell metabolism [12, 13] (Fig. 1).

### Fibroblast growth factor receptors and FGFs

The FGFR family comprises four FGFRs (FGFR1–4) and FGFRL1 that are all activated by specific FGFs [14, 15]. The 22 recognized FGFs are divided into three distinct functional groups—endocrine, paracrine/canonical, or intracellular—and further grouped into specific subfamilies based on their activity and sequence homology [16, 17, 18, 19, 20] (Table 1). FGFRs contain two or three immunoglobulin‐like domains (Ig‐like domains I, II, III) within their extracellular region, which can be alternatively spliced in FGFR1–3 to produce tissue‐specific “b” and “c” isoforms. The b and c isoforms vary at the C‐terminal half of Ig loop 3 [14]. The affinity between individual FGFs and each FGFR isoform enables the formation of a wide range of complexes and the regulation of differential cellular outputs [19, 21] (Table 1). Interaction of FGFRs with FGFs and the subsequent activation of FGFRs are regulated by co‐factors, such as heparin/heparin sulfate proteoglycans (HSPGs) or klotho proteins [22, 23, 24, 25]. Canonical FGFs are bound to HSPGs in the extracellular environment and this interaction is required to stabilize the ligand and the dimeric form of the receptor [26, 27, 28, 29]. HSPGs may also contribute to FGF signaling specificity. Endocrine FGFs, which have a weak affinity for HSPGs, require klotho proteins to facilitate their signaling [13, 29, 30]. Following specific ligand and co‐factor binding, each FGFR initially phosphorylates FGFR substrate 2α (FRS2α), which is constitutively associated with the receptor [31]. FRS2α then recruits adaptor proteins, such as growth receptor‐bound protein 2 (GRB2) and GRB2‐associated protein 1 (GAB1), to the activated receptor, which initiates the signaling cascades within the cell, including MAPK, PI3K, PLCγ, STATs [13, 19] (Fig. 1). Several negative signaling regulators, such as similar expression to FGF (SEF), GRB2, sprouty (SPRY), phosphatases like src homology‐2 protein tyrosine phosphatase (SHP2), and the E3 ubiquitin ligase CBL are integrated into the FGF/FGFR signaling network to attenuate signaling [32, 33, 34]. They often act together with noncanonical FGFR regulators, such as members of the cadherin, integrin, galectin, other RTK, and G‐protein‐coupled receptor (GPCR) families (Fig. 1).

**Table 1:** FGFs and their subfamilies, co‐factors (HSPGs or Klotho), FGFR isoforms, and tissue expression (at the RNA and/or protein level) according to the Human Protein Atlas (The Human Protein Atlas) and previous publications [13, 20, 39]. Each colour represents a different family of FGFs as in Figure 4.

Functional group	Subfamily	Co‐factor	FGFR	Tissue expression
**Endocrine FGFs**				
FGF15/19	FGF15/19	β‐klotho	1c, 2c, 3c, 4	Central nervous system (CNS), endocrine tissue, lung, digestive tract, gallbladder, pancreas, kidney, reproductive tissue, breast, heart, smooth and skeletal muscle, adipose tissue, skin
FGF21	FGF15/19	β‐klotho	1c and 3c	Liver, pancreas, testis
FGF23	FGF15/19	α‐klotho or β‐klotho	1c, 3c and 4	Retina, liver, heart, bone, thymus
**Paracrine/canonical FGFs**				
FGF1	FGF1	HSPGs	All FGFRs	CNS, kidney, breast, heart, liver, lung, skeleton, adipose tissue
FGF2	FGF1	HSPGs	1b, 1c, 2c, 3c, and 4	CNS, endocrine tissue, digestive tract, liver, kidney, reproductive tissue, breast, smooth muscle, adipose tissue, bone marrow, aorta, lung, skeleton
FGF4	FGF4	HSPGs	1c, 2c, 3c, and 4	Duodenum, ileum, skeletal muscle, adipose tissue, liver, colon
FGF5	FGF4	HSPGs	1c, 2c and 3c	CNS, gallbladder, kidney and smooth muscle, skin, hair, liver
FGF6	FGF4	HSPGs	1c, 2c, 3c, and 4	Skeletal muscle, tongue, adipose tissue
FGF3	FGF7	HSPGs	1b and 2b	CNS, eye, ear, lung
FGF7	FGF7	HSPGs	1b and 2b	Endocrine tissue, lung, digestive tract, gallbladder, bladder, reproductive tissue, breast, heart, smooth muscle, adipose tissue, skin, tongue
FGF10	FGF7	HSPGs	1b and 2b	Endocrine tissues, lung, digestive tract, gallbladder, pancreas, bladder, reproductive tissue, breast, white adipose tissue, skin, skeleton, urinary tract, CNS, liver
FGF22	FGF7	HSPGs	1b and 2b	CNS, reproductive tissues, skin
FGF9	FGF9	HSPGs	1c, 2c, 3b, 3c, and 4	CNS, retina, endocrine tissues, digestive tract, kidney, reproductive tissues, breast, heart, adipose tissue, brain, skeleton, lung, liver
FGF16	FGF9	HSPGs	1c, 2c, 3b, 3c, and 4	CNS, heart, olfactory bulb
FGF20	FGF9	HSPGs	1c, 2c, 3b, 3c, and 4	CNS, reproductive tissue, urinary tract
FGF8	FGF8	HSPGs	1c, 2c, 3b, 3c, and 4	Skeletal muscle, ovary, testes, CNS
FGF17	FGF8	HSPGs	1c, 2c, 3b, 3c and 5	CNS, endocrine tissues, skeleton
FGF18	FGF8	HSPGs	1c, 2c, 3b, 3c, and 6	Heart, skeletal muscle, adipose tissue, reproductive tissues, lung, endocrine tissues, brain, spinal cord, heart, skin
**Intracellular FGFs**				
FGF11	FGF11	Interact with NAv channels		CNS, retina, endocrine tissues, digestive tract, kidney, reproductive tissues, breast, heart, skeletal muscle, skin, lymphoid tissues, adipose tissue
FGF12	FGF11	Interact with NAv channels		CNS, endocrine tissues, retina, heart
FGF13	FGF11	Interact with NAv channels		CNS, endocrine tissues, retina, digestive tract, reproductive tissues, skeletal muscle, adipose tissue
FGF14	FGF11	Interact with NAv channels		CNS, endocrine tissues, retina, lung, digestive tract, reproductive tissues, heart

The FGF/FGFR signaling axis is a complex system regulated by various factors, including tissue localization, the nature and amount of the engaged FGF, specific phosphorylation patterns in the receptor tyrosine kinase domains, interactions with other cell surface molecules and receptors, and subcellular localization post‐internalization [19, 35, 36, 37, 38, 39, 40, 41] (Fig. 1). These factors work together to ensure tight regulation of downstream signaling, as aberrant activation of FGF/FGFR signaling is associated with developmental defects, metabolic disorders, and cancer [13, 16]. Here, we will focus on the role of the different FGF subfamilies in cellular metabolism. After an introduction on metabolism and cancer metabolism in particular, we will summarize what is known about the association of each FGF sub‐family with metabolism and will discuss implications for FGF signaling in cancer metabolism. Finally, we will suggest how the knowledge of FGF dysregulation in metabolism may lead to more effective treatments for cancer patients.

## Metabolism

Metabolism is a broad term used to summarize all the biochemical processes that occur within cells that allow them to grow, replicate, and respond to environmental cues [42]. Metabolic reactions encompass a wide range of pathways that are regulated by several enzymes and substrate‐led feedback mechanisms [43]. Throughout this review, we will focus on three main areas of whole‐body metabolism: glucose, lipid, and amino acid/protein metabolism. We will also introduce how signaling and metabolism are interconnected, specifically in the context of cancer metabolism.

### Glucose metabolism

**Fig. 2:**
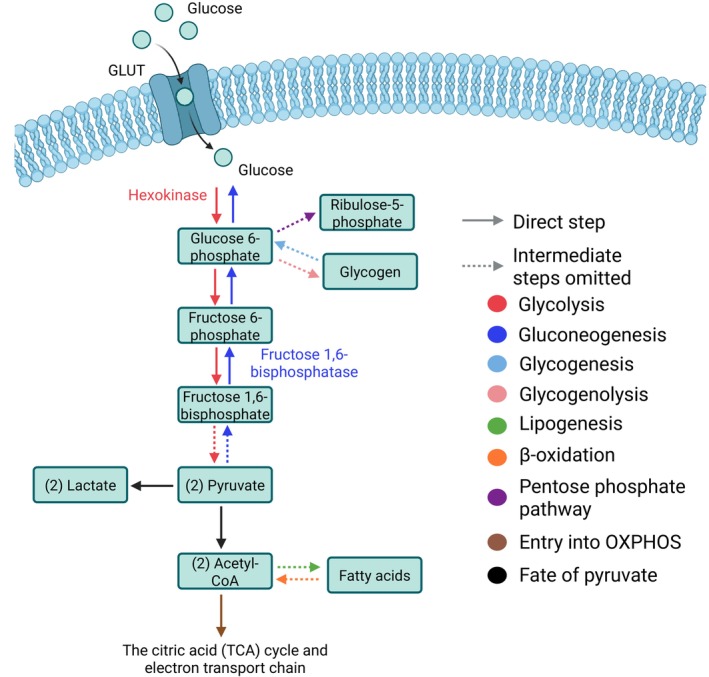
Schematic summarizing glucose metabolism. Created with BioRender (BH28QH1GQZ).

Glucose is the primary metabolic fuel in mammals [44]. It serves as a crucial component in many biological structures such as deoxyribose, galactose, and glycoproteins, as well as being the main source of energy for cells as it is broken down into ATP [44]. Glucose is derived from three main sources, either absorbed from digested carbohydrates, broken down from glycogen stores (throughout glycogenolysis), or made from non‐hexose precursors (throughout gluconeogenesis) [45] (Fig. 2). Glucose is transported into cells via glucose transporters (GLUTs) and is immediately phosphorylated to glucose‐6‐phosphate by hexokinases (HK) [46]. From here, glucose is either broken down in glycolysis and further within aerobic respiration to provide energy or within the pentose phosphate pathway (PPP) to fuel nicotinamide adenine dinucleotide phosphate (NADPH) and nucleotide synthesis, or stored as glycogen (summarized elsewhere [47, 48, 49, 50]) (Fig. 2). Following meals, β and α cells within the islets of Langerhans in the pancreas control the blood glucose levels within a tight range of 4–6 mm in humans by controlling the secretion of the opposing hormones insulin and glucagon [51, 52, 53]. Insulin docks to its receptor on muscle and adipose tissue to stimulate glucose uptake, as well as promoting glycogenesis in the liver, lipogenesis (synthesis of fatty acids from carbohydrate components), and incorporation of amino acids in proteins [54, 55, 56, 57, 58, 59]. In periods of fasting, the catabolic hormone glucagon is secreted from α cells in the pancreas to increase endogenous blood glucose levels by promoting hepatic glycogenolysis as well as hepatic and renal gluconeogenesis [51, 52, 60] (Fig. 2). Malfunction of the insulin‐glucagon axis leads to diabetes mellitus, a disease estimated to affect 5% of the global population [61]. Type 1 diabetes (T1DM) is characterized by the loss of β cells through autoimmunity and therefore the inability to produce insulin [51, 53]. Type 2 diabetes (T2DM) has a complex etiology, including exhaustion of β cells that prevents them from being able to meet functional insulin requirements, as well as insulin resistance in peripheral tissues [51, 53, 62]. T2DM is strongly affected by genetic components, and it is also often associated with obesity and age [63]. Obesity, which is mainly characterized by excessive fat accumulation in the body, also presents a high risk to health worldwide [64, 65]. Dysregulation of glucose metabolism is therefore associated with an increasing risk of cardiometabolic diseases, which affect a large part of the global population.

### Lipid metabolism

**Fig. 3:**
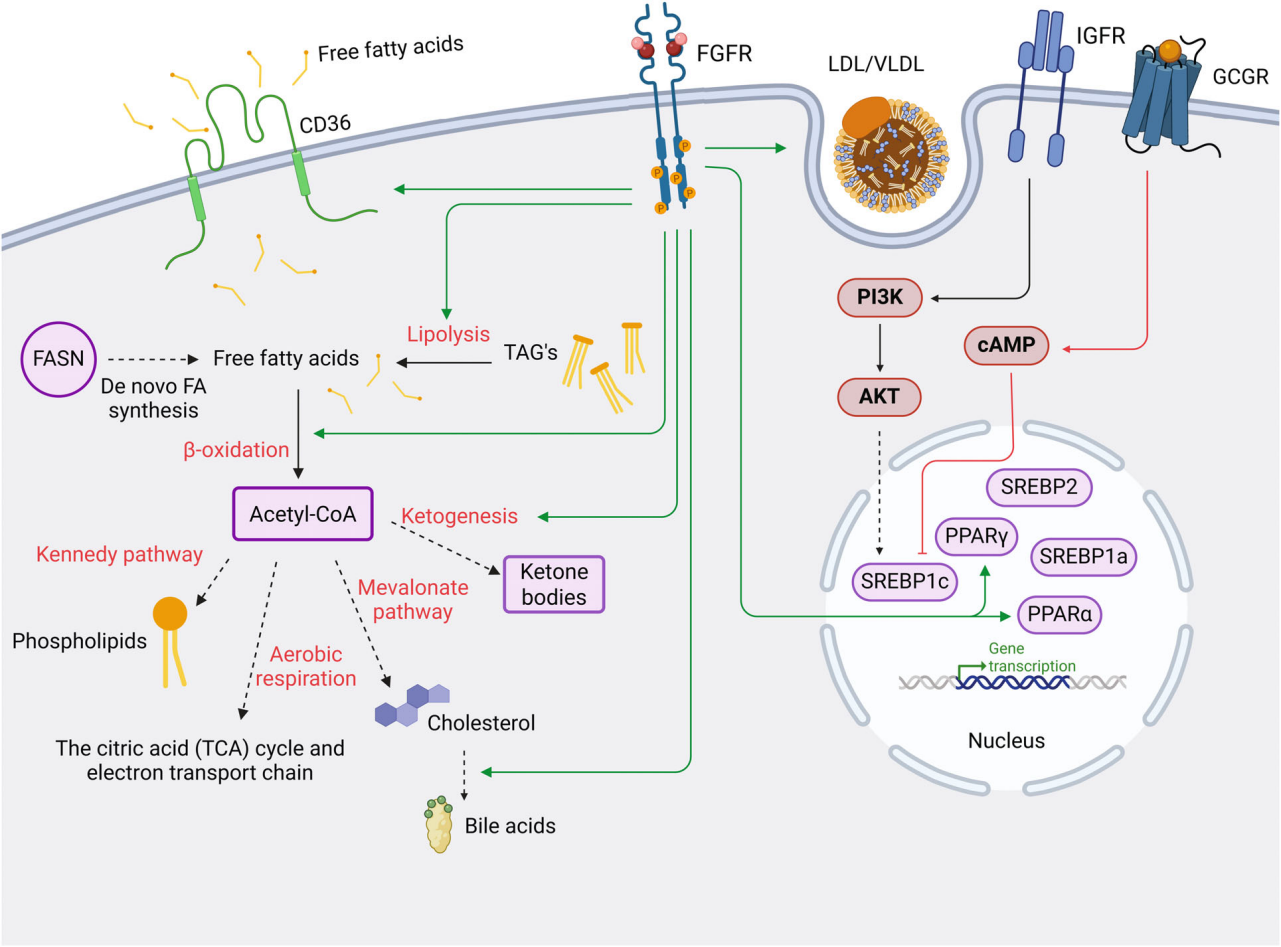
Schematic summarizing lipid metabolism, including the interplay between the glucose‐sensing hormones insulin and glucagon. The role of FGFR within lipid metabolism is also represented. GCGR, glucagon receptor; IGFR, insulin growth factor receptor; TAGs, triacylglycerols; VLDL, very low‐density lipoprotein. Created with BioRender (GM28RF9ZH0).

Lipids are absorbed from the diet where they are then packaged into chylomicrons or high‐density lipoproteins and transported to peripheral tissues, where they serve as a vital source of energy and are a key component of cell membranes [66, 67] (Fig. 3). When glucose levels are low, triacylglycerols (TAGs) are catabolized into fatty acids and can either undergo β‐oxidation to produce Acetyl‐CoA, which can enter the citric acid (TCA) cycle to generate energy or can be used to build membrane lipids such as phospholipids [66, 68]. Acetyl‐CoA can also be produced from glucose following glycolysis and can be used to synthesize new fatty acids, especially within the liver, adipose, and mammary tissue [69]. Acetyl‐CoA is the building block to generate cholesterol, another important membrane lipid, as well as ketone bodies for further energy storage [70] (Fig. 3). The sterol regulatory element‐binding protein (SREBP) transcription factors are known regulators of lipid biosynthesis, with SREBP1c mainly activating genes relating to fatty acid and triglyceride synthesis, SREBP2 regulating genes that control cholesterol synthesis, and SREBP1a activating genes in both pathways [71] (Fig. 3). Insulin transcriptionally induces SREBP1c through the PI3K pathway, whereas glucagon opposes this action through cyclic adenosine monophosphate (cAMP) [72, 73, 74] (Fig. 3), thus linking the regulation of glucose and lipid metabolism. The peroxisome proliferator‐activated receptors (PPARs) are other transcription factors that control lipid metabolism. PPARα is highly expressed in the liver, kidney, and muscle and controls genes involved in the oxidation and uptake of fatty acids and ketone body synthesis [75, 76, 77] (Fig. 3). Conversely, PPARγ controls genes that promote TAG synthesis and uptake of lipids in adipocytes [75]. Dysregulation of lipid metabolism is associated with obesity, diabetes, and cardiovascular disease [78].

### Amino acid and protein metabolism

Amino acids provide the building blocks of proteins, hormones, nucleotides, and neurotransmitters [79]. Amino acid‐dependent phosphorylation of mammalian target of rapamycin (mTOR), a crucial regulator of cellular metabolism, regulates protein synthesis [79, 80, 81, 82]. Hormones such as insulin, insulin‐like growth factor 1 (IGF‐1), and growth hormone (GF) also play a regulatory role in protein metabolism by decreasing protein degradation and promoting protein synthesis [83, 84, 85]. In contrast, glucagon stimulates an increase in hepatic amino acid uptake for gluconeogenesis and promotes protein breakdown, indirectly increasing glucose levels [86, 87]. Most amino acids, except for the nine essential amino acids (histidine, isoleucine, leucine, lysine, methionine, phenylalanine, threonine, tryptophan, and valine), can be derived from intermediates of core biological processes, such as glycolysis, TCA, and PPP, and can also be deaminated and fueled back into these pathways to supply energy [88, 89]. The metabolic pathway the amino acid enters dictates whether they are classified as either glucogenic or ketogenic amino acids [88]. The ammonia that is released following deamination of amino acids is converted to urea (the urea cycle) in the liver for excretion [90], a process that is lost in phenylketonuria and maple syrup urine disease [91]. Loss of regulation of amino acids and their products can result in neurological disorders, cancer, and cardiovascular diseases [79, 88].

### Signaling and metabolism

All the branches of metabolism work together to ensure that sufficient energy is supplied to cells. If intra‐regulatory mechanisms aren't sufficient to meet energy demands, the central regulator of cellular metabolism, AMP‐activated protein kinase (AMPK), is activated. AMPK senses depleted ATP levels and induces the phosphorylation of various substrates within all branches of metabolism to promote catabolic pathways and generation of ATP [92]. For instance, AMPK and mTOR are interconnected and regulate glucose, lipid, and amino acid metabolism, as well as autophagy and mitophagy [82, 92]. Another important regulator of cell metabolism is the PI3K‐Akt pathway, which regulates the metabolic changes downstream of insulin and controls cell proliferation, differentiation, and apoptosis [93, 94]. Besides the intracellular kinases AMPK and PI3K, RTKs and their ligands play important roles in metabolism regulation [95]. In particular, the endocrine ligands insulin and FGF21 and FGF19 have been studied in the context of glucose and lipid metabolism [10, 54]. More recently, RTKs other than insulin‐like growth factor receptor (IGFR), including epidermal growth factor receptor (EGFR) and the hepatocyte growth factor (HGF) receptor, c‐Met, have emerged as potential regulators of cell metabolism [11, 96, 97].

It is now accepted that not only is cell metabolism regulated by several signaling molecules, but also that certain metabolites, including lactate, regulate signaling cascades. This makes the reciprocal regulation of signaling and metabolism a central hub of cell function modulation with potential for novel therapeutic intervention in cancer as well as in cardiometabolic diseases [98, 99, 100, 101, 102].

### Rewired metabolism in cancer

Rewired metabolism within cancer has gained interest as a key factor in promoting growth and progression and is now recognized as one of the hallmarks of cancer [103, 104]. Cancer cells are known to favor glycolytic generation of ATP even in the presence of enough oxygen, a phenomenon known as the Warburg effect [105, 106, 107]. This metabolic switch allows cancer cells to produce excess glycolytic intermediates, which can fuel the biosynthesis of the macromolecules and organelles needed to sustain proliferation [108, 109]. Another hypothesis for this seemingly counterintuitive choice of ATP generation (only 2 ATP molecules are generated by one glucose molecule instead of the 28–32 ATP molecules generated by complete respiration utilizing oxidative phosphorylation) is that there is a symbiotic relationship between two populations of cells within cancer: one population creating a large volume of lactate as a byproduct of glycolysis and the other population utilizing this lactate as their main energy source [110, 111]. Cancer cells are also known to be dependent on high glutamine uptake, known as glutamine addiction [112]. Glutamine is used by cancer cells for amino acid biosynthesis, production of NADPH, and nucleotides, as well as promotion of mTOR activation and downstream protein translation [112, 113]. Furthermore, lipid metabolism is rewired in cancer, for instance, by upregulating fatty acid synthase (FASN), the major enzyme regulating fatty acid metabolism, which is now considered a novel target for cancer patients [114, 115, 116] (Fig. 3). Cancer cells also disrupt redox homeostasis to utilize reactive oxygen species (ROS) to promote tumorigenesis [117].

In addition to proliferation, rewiring of metabolism increases adaptation of cancer cells to hostile cancer microenvironments (e.g., in hypoxic conditions), the metastatic potential of cancer cells, immune escape, and resistance to therapies [118, 119, 120, 121]. Various metabolic diseases such as obesity and diabetes have also been linked to cancer development and cancer‐related mortality, and research continuously aims to explore the underlying molecular mechanisms [122, 123, 124]. Therefore, cancer metabolism and its regulation are an extremely important area of research to deepen the understanding of cancer progression and ultimately improve current cancer therapies.

## The FGF family

Within this review, we will provide insights into what is currently known about the role of FGFR ligands in metabolism regulation. In each subsection, we will also introduce the link between FGFR signaling and metabolism in the context of cancer. Finally, we will discuss the possible implications of inhibitors of FGFR signaling for regulating dysregulated cancer metabolism.

## Endocrine FGFs: FGF15/19, FGF21, FGF23

The endocrine or hormonal FGF subfamily consists of FGF15/19, FGF21, and FGF23 (Table 1). Due to their ability to enter the bloodstream and travel through the body, endocrine FGFs have been extensively characterized as metabolic regulators [125]. Here, we briefly summarize their known role in metabolism and refer the reader to an exhaustive recent review [12].

**Fig. 4:**
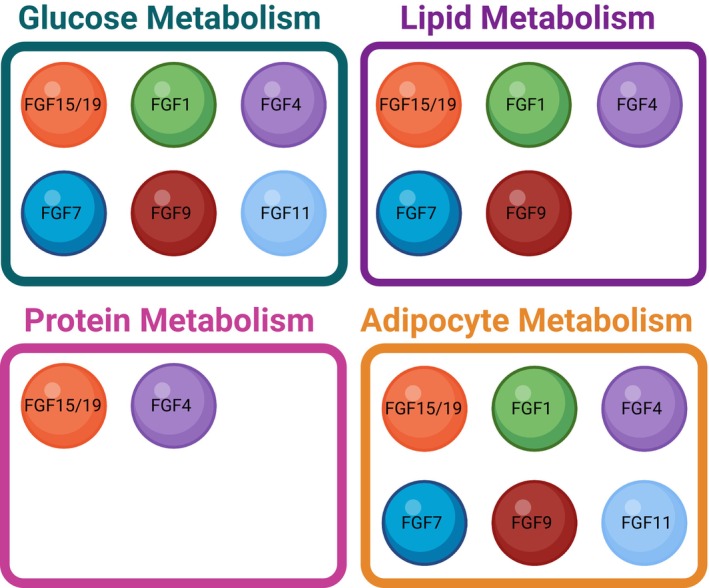
Schematic summarizing the branches of metabolism which have been associated with each subfamily of FGF. Created with BioRender (DP28GKBQIY).

FGF15 and FGF19 are orthologs of the same gene expressed in mice and humans, respectively [13]. FGF19 has been identified as a master regulator of bile acid synthesis (Figs 3 and 4). Following meals, bile acid is released into the intestine, where it emulsifies fats and facilitates lipid absorption [126]. In the intestine, bile activates farnesoid X receptor (FXR) to increase the transcription of FGF19 [127]. FGF19 then enters the bloodstream and, facilitated by βklotho and HS, activates FGFR4 in the liver [127]. FGF19‐FGFR4 regulates signaling pathways, including the c‐Jun N‐terminal kinase (JNK) and ERK, to repress the synthesis of bile acid from cholesterol, thereby reducing lipid absorption in the gut [128, 129]. Studies described weight loss following FGF19 administration in obese mice through FGF19‐dependent reduced lipid absorption from the diet [130]. Another mechanism through which FGF19 maintains lipid homeostasis occurs in the gut and in the liver, where FGF19 signaling regulates lipophagy and hepatic autophagy [131, 132] (Fig. 4). Furthermore, other studies showed that administration of FGF19 in obese diabetic mice improved blood glucose concentration through FGFR1c signaling in white adipose tissue (WAT) [10, 133] (Figs 2 and 4). Indeed, FGF19 may also contribute to maintaining glucose levels in an insulin‐independent manner in hepatocytes [134]. Through FGFR‐ERK‐ribosomal s6 kinase (R6K) signaling, FGF19 stimulates protein and glycogen synthesis and, through inactivation of cAMP, FGF19 decreases gluconeogenesis in the liver [134, 135]. Thus, FGF19 acts through multiple receptors and signaling pathways to regulate nutrient metabolism. On the same line, as FGFR4 and βklotho are expressed in the hypothalamus, FGF19 direct injection in the brain results in increased energy expenditure and decreased food intake in mice, and increased glucose disposal in rats [136, 137]. Furthermore, intracerebroventricular (i.c.v.) injection of FGF19, as well as of FGF1, in a T1DM rat model showed promising results for reversing diabetic symptoms by suppressing the hypothalamic–pituitary–adrenal (HPA) axis [138]. However, as FGF19 does not efficiently cross the blood–brain barrier, most of its effects are likely elicited through the peripheral tissue and not the CNS [139].

FGF21 is predominantly secreted from the liver and is a master regulator of glucose and lipid metabolism [140] (Fig. 4). During periods of fasting, PPARα induces the expression and secretion of FGF21 from the liver, which coordinates a switch from using carbohydrates as the main energy source to utilizing fatty acids [141]. This may open an interesting avenue of research for FGF21 within cancer as lipidomic remodeling is well characterized within tumors, leaving cancer cells reliant on fatty acid oxidation for energy production [142]. Additionally, the role of PPARs in cancer progression is currently quite controversial, as it is tumor‐ and context‐dependent and regulates angiogenesis, proliferation, and the tumor microenvironment [143, 144]. Perhaps the PPARα‐FGF21 axis could be further explored in cancer metabolism, given that the role of FGF21 within cancer metabolic rewiring is understudied. Tumors secrete FGF21 to sustain AKT‐mTOR1‐SREBP1 signaling in CD8^+^T to rewire their cholesterol metabolism and impair their function and facilitate immune invasion [145]. Tumor‐secreted FGF21 may also regulate cancer cell metabolism in an autocrine manner. As FGF21 analogues and mimetics are currently in clinical trials for the treatment of various metabolic disorders such as metabolic syndrome‐associated steatohepatitis (MASH), this may open novel opportunities to modulate cancer metabolism [146, 147]. FGF21 seems also to act as a starvation hormone to induce fatty acid oxidation, gluconeogenesis, and ketogenesis by inducing an increase in the expression of PPARγ coactivator protein‐1α (PGC‐1α) [148]. In a fed state, FGF21 can be released from adipose tissue, where it acts in an autocrine manner [149]. Within adipose tissue, FGF21 stimulates insulin‐dependent glucose uptake, enhances PPARγ activity, and stimulates the release of adiponectin, an adipokine well characterized for its metabolic role in controlling systemic glucose and lipid homeostasis [149, 150, 151, 152]. Some studies even argue that the positive downstream effects of FGF21 on metabolism are in fact the actions of adiponectin [152, 153]. How FGF21 controls lipolysis is also controversial, with some studies showing FGF21 signals through FGFR1‐β klotho to limit lipolysis and others showing it induces lipolysis in WAT [141, 154, 155]. FGF21 also stimulates the browning of WAT through the actions of PGC‐1α, which leads to increased energy expenditure and glucose uptake [156, 157, 158]. The increase in energy usage may also be mediated by FGF21‐dependent activation of liver kinase B1 (LKB1), which in turn activates downstream signaling to enhance mitochondrial oxidative phosphorylation [159]. The increased glucose uptake in WAT is hypothesized to be a result of ERK‐transcription factor Elk‐1‐serum response factor (SRF) signaling stimulating an increase in GLUT1 expression [150, 160]. In studies on diabetic and obese mice, FGF21 is found to induce whole‐body insulin sensitization and a potent antihyperglycemic effect, as well stimulate a decrease in hepatic and plasma triglyceride levels, an increase in energy expenditure, and consequently significant weight loss [150, 161, 162, 163]. The molecular determinants of this effect of FGF21 on nutrient metabolism have been ascribed to stimulation of the secretion of insulin from β cells of the pancreas through ERK1/2 signaling and to FGF21‐dependent activation of the AKT‐BAD signaling axis to protect pancreatic islets from glucolipotoxicity and apoptosis, with a consequent increase in islet numbers and preservation of β cell function [164]. Unlike FGF19, FGF21 can enter the brain and can control metabolism through direct actions on the CNS [165]. For instance, FGF21 signaling in tanycytes enables free fatty acid sensing, implicating FGF21 in central regulation of lipid metabolism [166]. In line with this idea, FGF21 stimulates the secretion of adrenal glucocorticoids to promote hepatic gluconeogenesis as part of the adaptive starvation response, and i.c.v. infusion of FGF21 in obese rats results in insulin‐induced suppression of both hepatic glucose production and gluconeogenic gene expression [167, 168]. Overall, FGF21 has a multifaceted and crucial role in metabolism regulation, with its effects dysregulated in metabolic disorders such as diabetes and obesity, pointing to FGF21 as a potential new candidate to modulate cancer metabolism.

FGF23 is synthesized and secreted by osteocytes and osteoblasts, and controls mineral and vitamin D homeostasis through actions in the kidney and parathyroid gland [169]. FGF23 inhibits phosphate reabsorption and vitamin D synthesis in the renal proximal tubules and inhibits parathyroid hormone production in the parathyroid gland to control calcium levels [169, 170, 171]. Its metabolic effects are therefore quite specific compared to those of FGF21 and FGF9.

## Paracrine FGFs: FGF1, 4, 7, 8, 9 subfamilies

The paracrine/canonical FGFs have a high affinity for HSPGs, which are ubiquitously expressed throughout the body within the extracellular matrix or cell membranes [172]. This tight interaction results in paracrine FGFs being contained within the matrix of the secreting cells, and their actions are elicited only locally [173]. HSPGs stabilize dimerization and activation of FGFRs to promote mitogenic and angiogenic signals [36]. The angiogenic and mitogenic potential of the canonical FGFs is well documented [13]. However, they have recently gained attention for a potential role within metabolism as well [12].

### FGF1 subfamily: FGF1, FGF2

FGF1 is unique among FGFs in its ability to bind and activate all FGFR isoforms (Table 1). FGF1 is expressed in several metabolic tissues, including the liver, kidney, brain, and WAT, where its expression is significantly upregulated in obese patients [10, 174]. The metabolic activity of FGF1 has been extensively characterized. For instance, PPARγ and Piezo1 stimulate an increase in FGF1 levels in adipocytes of mice following a high‐fat diet [175, 176] (Fig. 4). FGF1 then regulates adipose tissue remodeling and pre‐adipocyte differentiation and enhances insulin sensitivity to restore glucose levels [175, 176, 177, 178]. FGF1 is also seen to improve insulin resistance by decreasing adipose tissue inflammation through mTOR signaling [179]. The action of FGF1 on adipocytes is also relevant within breast cancer, where FGF1 stimulates cancer cell proliferation and plays a role in the reprogramming of metabolism through activation of the estrogen receptor (ER) in the ER‐positive breast cancer subtype [180, 181]. Particularly in ER‐positive breast cancer patients, obesity induces the expression of FGF1, which, through the activity of FGFR1, can aid progression and growth as well as therapy resistance [182]. This is an outstanding example of how mitogenic FGFs with an emerging role in metabolism may contribute to cancer in two ways: by promoting cell proliferation and by controlling cell metabolism. Interestingly, the angiogenic potential of FGF1 to stimulate vascularization within adipose tissue, contributing to its remodeling, as well as within cancer cells, suggests that FGF1 may control the cancer microenvironment by acting on both cancer cells and adipocytes [183, 184]. Further studies in obese and diabetic mice have explored the antihyperglycemic role of FGF1 following acute and chronic administrations of FGF1 and identified direct actions of FGF1 through FGFR1 in the WAT [185, 186] (Fig. 4). Within 3T3‐L1 adipocytes, acute FGF1 stimulation increases the activation of GLUT4 through crosstalk between MEK1/2 and AKT, whereas chronic administration of FGF1 increases the expression of GLUT1 through MEK1/2 [185]. Chronic FGF1 administration in obese and diabetic mice also stimulates systemic enhancement of insulin sensitivity with decreased liver glycogen storage and steatosis and increased glucose uptake in skeletal muscles through increased expression of GLUT4 [186, 187]. Therefore, FGF1‐dependent potential regulation of GLUT levels in several cell types, including epithelial cancer cells, may contribute to the reciprocal regulation of signaling and metabolism in the context of human cancer. Another mechanism through which FGF1 stimulates a reduction in glucose production is by activating phosphodiesterase 4D (PDE4D) in adipocytes via the FGFR1‐PI3K signaling axis to inhibit lipolysis [188, 189]. Within the CNS, FGF1 levels increase postprandially and have been reported to suppress food intake to directly regulate consumption, unlike FGF21, which controls metabolism downstream [190]. As mentioned above, i.c.v. injections of both FGF1 and FGF19 also improve glucose metabolism in diabetic rodents, highlighting that both endocrine and paracrine FGFs can centrally control glucose metabolism [138].

FGF2 signaling also decreases glucose levels, potentially through its actions within adipocytes, where it stimulates an increase in GLUT1 expression and in glycolysis, as well as lipid levels and low and very low‐density lipoproteins in diabetic rats [191, 192]. The role of FGF2 within adipose tissue metabolism is, however, controversial. One study demonstrates that FGF2 decreases uncoupling protein 1 (UCP1) expression through inhibition of PGC‐1α and PPARγ to ultimately decrease energy expenditure in brown adipose tissue (BAT) and WAT [193]. Other studies show instead that the administration of FGF2 increases UCP1 and thermogenesis [194] There are also contrasting results regarding FGF2 adipogenic properties [195, 196]. FGF2 has been identified to play a crucial role in the self‐renewal of adipocyte‐derived stem cells, as well as in stimulating proliferation through EKR signaling [197]. Most likely, FGF2 functions are cell‐ and tissue‐dependent, and more work needs to be done to link FGF2 to cell and, potentially, cancer metabolism. However, as FGF2 regulates neovascularization by increasing the expression of the glycolysis enzyme HK2 and by promoting the proliferation and migration of endothelial cells, FGF2 may synergize with FGF1 in controlling vascularization in the cancer microenvironment [198].

### FGF4 subfamily: FGF4, FGF5, and FGF6

FGF4 has the same antihyperglycemic properties as FGF1 following i.c.v. injection into diabetic mice and is being explored as a possible treatment for T2DM [199]. Similarly to FGF1, FGF4 stimulates an increase in GLUT4 expression in skeletal muscle in an AMPKα‐dependent manner to decrease blood glucose levels [187] (Fig. 4). Chronic treatment of diabetic mice with FGF4 resulted in an anti‐inflammatory response in adipose tissue and an overall increase in insulin sensitivity [187]. FGF4 activates hepatic FGFR4 and AMPK and stimulates enhanced fatty acid oxidation and reduced apoptosis [200]. Therefore, FGF4 is a candidate in the rewiring of metabolism, especially as fatty acid oxidation in cancer cells protects them from apoptosis, contributing to chemoresistance [201]. Of note, FGFR4 regulates lipid metabolism in triple‐negative breast cancer to promote proliferation and invasion [202]. It would be interesting to link this to the effect of FGF4 or other FGFR4 ligands. A non‐mitogenic analogue of FGF4 is now being explored as a possible therapeutic candidate for the treatment of nonalcoholic fatty liver disease (NAFLD) [203]. Similarly to FGF1, FGF4 mitogenic and metabolic activities seem to depend on tissue type (e.g., different effects of FGF1 and FGF4 on CNS, adipose tissue, and hepatocytes) and on the expressed genome (e.g., they act either through FGFR1 and FGFR4). FGF4 is also reported to work alongside FGF19 to control bile acid synthesis in a paracrine manner [204].

FGF5 was recently found to ameliorate hepatocyte apoptosis in mouse models of liver injury through the PI3K‐Akt pathway, similarly to FGF4 [200, 203, 205]. However, its action on metabolism remains unexplored and is worth studying further.

FGF6, as well as FGF9, activates FGFR3 and stimulates a downstream increase in UCP1 expression in adipocytes and pre‐adipocytes following exercise or cold exposure [206]. FGF6 also contributes to adipose tissue metabolism by acting as a proliferative factor on adipocyte progenitor cells to increase insulin sensitivity and maintain fat homeostasis [207]. Finally, FGF6 administration into the skeletal muscle of obese mice stimulates protein synthesis via the mTOR pathway and prevents weight gain and insulin resistance [208]. Through all these mechanisms, FGF6 mainly controls energy homeostasis in adipose tissue in the context of metabolism regulation.

### FGF7 subfamily: FGF3, FGF7, FGF10, and FGF22

FGF3 and FGF22 are not well studied in the context of cellular metabolism, although there is evidence implicating FGF3 in diabetes and its consequences, such as diabetic retinopathy [209]. However, further investigation is needed to fully understand the role of FGF3 in metabolism. FGF22 has predominantly been studied in relation to its function in neural development and synapse formation [13, 210]. Thus, FGF22 does not currently have any well‐established roles within metabolism.

FGF7 and FGF10 bind the epithelial b isoforms of FGFR1 and 2, with FGF7 being more specific for FGFR2b (Table 1); they are potent mitogens that play crucial roles, among others, in wound healing, development, and cancer, including breast cancer [16, 211, 212, 213]. In the context of cellular metabolism, the role of FGF7 and FGF10 is beginning to emerge (Fig. 4). Indeed, FGF7 has been found to ameliorate diabetic symptoms with promising effects in the healing of diabetic wounds and following islet transplantation [214, 215]. Oral administration of FGF7 is also used to treat oral mucositis caused by chemotherapy following hematologic cancer [216]. This makes FGF7 a potential candidate worth exploring for the treatment of wound healing in metabolic and cancer diseases. FGF7 has also been shown to exacerbate COVID‐19 infection in pancreatic islet cells by increasing the expression of angiotensin‐converting enzyme 2 (ACE2), the membrane receptor necessary for viral attachment and entry [217]. ACE upregulation also results in increased insulin secretion [217, 218]. This shows an interesting, still unexplored, connection between glucose metabolism and COVID‐19 infection. Future studies could focus on analyzing the connection between FGF7 and physiological metabolism within pancreatic islets or alveolar cells in the lung, where both ACE and FGFR2b are expressed.

FGF10 regulates adipocyte metabolism within WAT by stimulating an increase in lipoprotein lipase (LPL), an enzyme that hydrolyzes lipoproteins to release triglycerides, as well as by stimulating pre‐adipocyte proliferation and adipogenesis through the activation of the Ras/MAPK pathway and downstream transcription factors [219, 220]. If these roles of FGF10 on adipocytes are confirmed in breast adipocytes, this may open novel avenues to explore in the context of how cells in the breast cancer microenvironment (e.g., adipocytes) affect tumorigenesis. Indeed, FGF10 is overexpressed in breast cancer and signals through its receptor FGFR2b in breast cancer epithelial cells [16]. Although direct roles for FGF10 in cancer metabolism have not been reported to the best of our knowledge, we have recently reported that the FGF10‐FGFR2b signaling axis regulates AMPK phosphorylation, prevents autophagy, and promotes cell survival through regulation of mTOR signaling and unc‐51 like autophagy activating kinase 1 (ULK1) phosphorylation in breast cancer cell models [221]. These data suggest a link between FGF10 signaling, cell survival, and the regulation of energy metabolism. Along this line, FGF10 stimulates AMPK activation in the liver, thus promoting lipolysis and diminishing lipid accumulation [222]. Finally, FGF10‐expressing tanycytes in the CNS have been found to generate parenchymal neurons with roles in regulating appetite and energy balance [223].

### FGF8 subfamily: FGF8, FGF17, and FGF18

Members of the FGF8 family are not reported to have any metabolic activity except for FGF17, which may have antagonistic properties to FGF19 in the hypothalamus in relation to glucose homeostasis [224].

### FGF9 subfamily: FGF9, FGF16 and FGF20

We will focus on FGF9 and FGF16 as the metabolic role of FGF20 hasn't been explored yet.

In diet‐induced obese mice, overexpression of FGF9 in the liver alleviates hepatic steatosis, improves insulin sensitivity, and decreases glucose levels by inhibiting lipogenesis and increasing fatty acid oxidation [225] (Fig. 4). The role of FGF9 in adipose tissue is, however, controversial. Whereas some studies report that FGF9 controls thermogenesis and browning of WAT in mice through the increase of UCP1, others claim that FGF9 has the opposite effect in adipose tissue, with FGF9 and hypoxia signaling preventing the browning of adipocytes [194, 206, 226]. Based on FGF9‐dependent control of metabolism, a role for FGF9 in cancer metabolism, specifically of ovarian cancer, has been hypothesized. Indeed, FGF9 signaling has been associated with the lethality of ovarian cancer through the control of metabolic reprogramming and downstream increase in metastatic potential [227]. In this context, FGF9 regulates the increase in aerobic glycolysis and the activation of the transcription factor C‐ets‐1 (ETS1), both stimulating an increase in vascular endothelial factor‐A (VEGF‐A)/VEGF receptor 2 expression and subsequent enhancement of angiogenesis and invasiveness [227].

Similarly to FGF9, FGF16 has been recently reported to induce a shift to aerobic glycolysis in breast cancer cells, thus promoting cellular invasion [228]. Independent studies in mice have found FGF16 to be a metabolic regulator that improves glucose metabolism and weight loss [229]. FGF16 is seen to induce UCP1 in WAT; however, the downstream weight loss is not a result of increased energy expenditure but is seen to be due to reduced food and water intake and absorption [229].

## Intracellular FGFs: FGF11, FGF12, FGF13, and FGF14

The intracellular FGFs (iFGFs) are non‐signaling proteins that act as cofactors for voltage‐gated sodium channels and other molecules and are essential regulators of neuronal and myocardial excitability [14, 230]. However, the role of a few members of the iFGFs family in metabolism has very recently begun to emerge.

For instance, hypothalamic FGF11 can centrally control metabolism by regulating the expression of neuropeptide Y (NPY) [231]. Indeed, FGF11 knockdown in mice leads to a decrease in NPY expression, which in turn ameliorates the effects of a high‐fat diet, including decreased weight gain, increased BAT thermogenesis, and improved glucose and insulin intolerance [231] (Fig. 4).

FGF13 also seems to play a role in metabolism. FGF13 expression in adipose tissue is increased in obese mice and in humans and is found to impair glucose utilization and thermogenesis, dysregulating energy and glucose homeostasis [232]. FGF13 has also been recognized as a potential biomarker of T2DM and worsens diabetic glomerular injury, a kidney disease that can occur in diabetic patients [233, 234]. In the context of cancer metabolism, the long noncoding RNA lncRNA FGF13‐anti‐silencing 1 (FGF13‐AS1) is downregulated in breast cancer patients [235]. Similarly to mRNA, lncRNAs are important regulators of gene expression and can regulate a multitude of different cellular processes such as proliferation and metabolism [236]. *In vitro* experiments found FGF13‐AS1 impair glycolysis and stemness properties, resulting in decreased proliferation, migration, and invasion [235]. Therefore, we can hypothesize a role for FGF13 in rewiring metabolism, specifically glucose metabolism, within cancer to promote progression.

In contrast, the role of FGF12 in metabolism has not been reported yet. However, FGF12 has been linked to cancer progression, cardiac diseases, and nervous system disorders [237]. Interestingly, FGF12 is secreted from cells despite lacking the canonical signal for secretion [238]. Therefore, FGF12 can signal through FGFRs and control apoptosis and ribosome biogenesis [239, 240]. Based on such recent developments uncovering FGF12 signaling capabilities, the future involvement of FGF12 within metabolism may be revealed in different cell types.

Similarly to FGF12, FGF14 activity has been well characterized in excitable neuronal cells, and overexpression in cancer—specifically lung and pancreatic—has been associated with worse prognosis for patients [241, 242]. However, no metabolic functions have been reported for FGF14 to the best of our knowledge.

## Conclusions and perspectives

As FGF signaling plays a crucial role both within metabolism and tumorigenesis, it is time to speculate that FGF signaling may be a master regulator of cancer metabolism [20].

Metabolic reprogramming within cancer is necessary to provide sufficient energy and biomolecules to sustain uncontrolled growth [103]. The unique cancer metabolism encompasses dysregulation of various metabolic processes, such as increased anaerobic glycolysis, fatty acid oxidation, and glutamine catabolism to meet energy demands and fuel macromolecular synthesis [103, 112, 142, 243] (Figs 2 and 3). The metabolism of cancer cells is recognized as being context specific, with tissue origin and stage of cancer having the strongest influence. For example, pancreatic cancer cells may become more reliant on OXPHOS in advanced stages to promote cancer progression [244]. Studies show that transformed cells maintain and manipulate similar metabolic profiles as the tissue of origin. For instance, within tumors of the liver and kidney, the gluconeogenic enzyme fructose‐1,6‐bisphosphatase 1 (FBP1) is lost to promote glycolysis [245, 246]. Along this line, tissues where FGF signaling controls cellular metabolism (Table 1) could be targeted with FGF signaling analogues or inhibitors to alter cancer metabolism.

Rewired metabolism is also advantageous to cancer cells for facilitating the metastasis process. For cancer cells to disseminate, they must overcome a plethora of challenges [247, 248]. In the early stages of the metastasis process, the excess lactate, CO_2_, and other organic acids produced by cancer cells acidify the extracellular matrix (ECM) and aid its degradation [243, 249]. FGF/FGFR signaling within cancer, such as prostate cancer, dysregulates the expression of lactate dehydrogenase (LDH) enzymes to increase lactate production and favor motility [250]. Once cancer cells are detached from the ECM, they need to infiltrate and survive within the circulation. The PI3K‐Akt signaling axis is reported to increase NADPH and glutathione (GSH) in various cancers [251]. These vital antioxidant molecules are repurposed in cancer as protection against oxidative stress due to mass proliferation or within the circulation during metastasis [252, 253]. FGF signaling may contribute to this process by controlling angiogenic potential, neovascularization within tumors, and anti‐oxidative effects [183]. Finally, anchoring and colonization of a distant organ also require metabolic flexibility, as the new environment may have different metabolic requirements [247, 254]. For instance, it has been recently shown that metastasizing breast cancer cells activate pyruvate carboxylase‐driven metabolic pathways in response to the lung microenvironment [255]. Abnormal adhesion properties are required for efficient metastasis, and RTKs, such as FGFR and EGFR, are found in complex with adhesion molecules, such as integrins and neural cell adhesion molecule (NCAM) [256, 257, 258]. As dysregulated RTK signaling has been associated with several of the mentioned metastasis steps, RTK inhibitors may be useful not only to inhibit cancer cell proliferation and motility but also to revert cancer metabolism underlying tumorigenesis and metastasis. For instance, metabolic vulnerabilities of FGFR and EGFR have been studied in the context of lung cancer [259].

The unique metabolic properties of cancer cells also contribute to acquired therapy resistance. For instance, in non‐small‐cell lung cancer treated with the EGFR inhibitor erlotinib, lactate released from the cancer cells induces neighboring fibroblasts to release HGF to sustain oncogenic signaling through the activation of cMET on cancer cells, making EGFR inhibition ineffective [260].

The recently described role of RTKs, including EGFR and cMET, within cancer metabolism presents an interesting area of research to uncover new biomarkers or drug targets in cancer treatment [11, 96]. However, heterogeneity constitutes the main challenge with targeting cancer metabolism. Cancer subtype and tissue localization affect the specific metabolism within tumors [261]. Also, very few metabolic processes are confined to tumors only. Therefore, the off‐target effects of potential therapies targeting cancer metabolism need to be closely considered. With a more complete understanding of whole‐body metabolism and the specific metabolism of each cancer type, as well as with a better understanding of how RTK signaling is implicated in cancer metabolism, target therapies may be combined with specific diets, such as intermittent fasting or ketogenic diets, to overcome the toxic side effects of signaling and other cancer molecule inhibitors [262, 263, 264].

Ongoing research continues to explore FGF signaling, predominantly endocrine FGFs, as treatments for various metabolic diseases; with the huge influence that obesity and diabetes have on cancer rates and mortality, this research may prove beneficial for understanding how FGF signaling contributes to cancer metabolism [122, 123, 124].

In summary, we have described the regulatory role of FGF signaling in different metabolic pathways, and we have discussed the enormous potential of using inhibitors against FGFR and other RTK signaling in the context of novel targeted therapies against cancer and cancer metabolism.

## Author contributions

JP wrote the manuscript and prepared the figures. CF supervised JP and wrote the manuscript. All the authors approved the manuscript before submission.
